# Heterogeneity in convergence behaviour of the single-step SNP-BLUP model across different effects and animal groups

**DOI:** 10.1186/s12711-023-00856-5

**Published:** 2023-11-23

**Authors:** Dawid Słomian, Kacper Żukowski, Joanna Szyda

**Affiliations:** 1 National Research Institute of Animal Production, Krakowska 1, 32-083 Balice, Poland; 2 Biostatistics Group, Department of Genetics, the Wroclaw University of Environmental and Life Sciences, Kozuchowska 7, 51-631 Wroclaw, Poland

## Abstract

**Background:**

The single-step model is becoming increasingly popular for national genetic evaluations of dairy cattle due to the benefits that it offers such as joint breeding value estimation for genotyped and ungenotyped animals. However, the complexity of the model due to a large number of correlated effects can lead to significant computational challenges, especially in terms of accuracy and efficiency of the preconditioned conjugate gradient method used for the estimation. The aim of this study was to investigate the effect of pedigree depth on the model's overall convergence rate as well as on the convergence of different components of the model, in the context of the single-step single nucleotide polymorphism best linear unbiased prediction (SNP-BLUP) model.

**Results:**

The results demonstrate that the dataset with a truncated pedigree converged twice as fast as the full dataset. Still, both datasets showed very high Pearson correlations between predicted breeding values. In addition, by comparing the top 50 bulls between the two datasets we found a high correlation between their rankings. We also analysed the specific convergence patterns underlying different animal groups and model effects, which revealed heterogeneity in convergence behaviour. Effects of SNPs converged the fastest while those of genetic groups converged the slowest, which reflects the difference in information content available in the dataset for those effects. Pre-selection criteria for the SNP set based on minor allele frequency had no impact on either the rate or pattern of their convergence. Among different groups of individuals, genotyped animals with phenotype data converged the fastest, while non-genotyped animals without own records required the largest number of iterations.

**Conclusions:**

We conclude that pedigree structure markedly impacts the convergence rate of the optimisation which is more efficient for the truncated than for the full dataset.

## Background

The single-step model will soon become the standard procedure of most national genetic evaluations of dairy cattle [1, 2]. In spite of its great advantages for routine evaluations, with the most important being the possibility of conducting a joint breeding value estimation for ungenotyped and genotyped individuals, it should be kept in mind that statistically it is a very highly parameterised model that involves the estimation of several millions of effects that are often highly correlated. This poses potential problems in solving the system of equations, and most implementations have used the preconditioned conjugate gradient method (PCG) to solve these sets of equations. The PCG method was introduced for genetic evaluation models by Strandén and Lidauer in 1999 [3], which was further developed by Vandenplas et al. [4, 5] in the context of the single-step single nucleotide polymorphism best linear unbiased prediction (SNP-BLUP) model. Still, as indicated by these authors, especially in the context of the single-step SNP-BLUP model, a fast rate of convergence of the PCG requires an additional, second-level preconditioner. Moreover, on the national scale, the model is applied to very large datasets consisting of millions of records, which makes the solving technically and computationally demanding, not only in the context of memory consumption and CPU usage but also in the context of the numerical accuracy of calculations that is especially pronounced in parallel applications of PCG [6]. Relating to the above, the goal of our study was twofold: (1) to examine the differences in the model convergence depending on the number of individuals considered in the evaluation; and (2) to examine the convergence rate for different components of the model.

## Methods

### Materials

The analyzed dataset (Table 1) corresponds to the Polish national genetic evaluation for stature from December 2021 and comprised 1,098,611 cows with phenotypes for stature and 141,397 bulls with pseudo-phenotypes expressed by their de-regressed proofs (DRP) from the multiple across country evaluation (MACE) carried out by Interbull (interbull.org). Full genomic data in the form of genotypes of 46,118 SNPs were available for 134,960 individuals, including 70,134 cows with phenotypes as well as 64,826 bulls among which 26,471 were young individuals without pseudo-phenotypes and 38,355 were bulls with MACE-DRP. The majority of the genotyped individuals were genotyped using various versions of the EuroG MD Illumina genotyping microarray, containing more than 45,000 SNPs, that was customized for the EuroGenomics Cooperative. Individuals genotyped with other commercial platforms were imputed to EuroG MD using the Fimpute software [7]. In addition to the standard set of the 46,118 SNPs, for which a minor allele frequency (MAF) of 0.0064 corresponded to that of the genomic data used in the routine genomic evaluation, three SNP sub-sets, selected from this standard set, were considered based on MAF: (1) 45,537 SNPs with a MAF ≥ 0.01, (2) 41,667 SNPs with a MAF ≥ 0.05, and (3) 37,380 SNPs with a MAF ≥ 0.1. Furthermore, two strategies for the use of pedigree information were considered: (1) using the pedigree data for all available ancestors, i.e. 8,461,877 animals and 36 genetic groups (FULL); and (2) using the pedigree data for animals with phenotype or genotype data truncated after the fifth generation, which resulted in 1,555,995 individuals and 33 genetic groups (5GEN). Genotype data and pedigree information were stored in the cSNP database maintained by the National Research Institute of Animal Production [8].

**Table 1:** Numbers of animals in the analysed datasets

Category	Sex	Number of animals
Phenotype data	Females with phenotypes	1,098,611
	Males with MACE DRP	141,397
Genotype data	Females	70,134
	Males (bulls and candidates)	64,826
Pedigree data		
All generations	Females	6,428,481
	Males	2,023,328
5th generations	Females	1,368,487
	Males	187,508

### Methods

The following single-step SNP-BLUP model [9] was considered:

1 $$\mathbf{y}=\mathbf{X}{\varvec{\upbeta}}+\mathbf{W}\mathbf{a}+\mathbf{e},$$

where $\mathbf{y}$ is the vector of dependent variables represented by the cows’ measured phenotypes for stature and bulls' pseudo-phenotypes expressed by their MACE DRP, ${\varvec{\upbeta}}$ is the vector of fixed effects including age at calving, lactation phase, and herd corresponding to the cows’ phenotypes and bulls’ DRP (note that for the bulls artificial codes for fixed effect class were used), $\mathbf{a}$ is the vector of the individuals’ breeding values and genetic groups, which for the genotyped part of the population is expressed as $\mathbf{a}=\mathbf{Z}\mathbf{g}+\mathbf{u}$ with $\mathbf{g}$ being the vector of random SNP effects, $\mathbf{u}$ is the vector of random additive (residual) polygenic effects, $\mathbf{e}$ is the vector of residuals, $\mathbf{X}$, $\mathbf{Z}$ and $\mathbf{W}$ are the corresponding design/incidence matrices. Genetic groups were defined based on a 10-year window based on the year of birth of the animals, separately for cows, bulls and their country of origin provided by: Poland, USA-Canada, and the remaining countries. The underlying covariance structure of the model is given by: $\mathbf{g}\sim \mathrm{MVN}\left(\mathbf{0},{\mathbf{I}\cdot \frac{1-\mathrm{k}}{2\sum_{\mathrm{i}=1}^{\mathrm{N}}{\mathrm{p}}_{\mathrm{i}}\left(1-{\mathrm{p}}_{\mathrm{i}}\right)}\upsigma }_{\mathrm{a}}^{2}\right)$, $\mathbf{u}\sim \mathrm{N}\left(\mathbf{0},{\mathbf{A}\cdot \mathrm{k\sigma }}_{\mathrm{a}}^{2}\right)$, and $\mathbf{e}\sim \mathrm{N}\left(\mathbf{0},{\mathbf{D}\upsigma }_{\mathrm{e}}^{2}\right)$, where $\mathrm{k }\left(=0.2\right)$ corresponds to the proportion of additive genetic variance due to the residual additive polygenic effect i.e. not explained by SNP genotype variation, ${\mathrm{p}}_{\mathrm{i}}$ is the frequency of allele *A* of the ith SNP of N SNPs, $\mathbf{A}$ is the numerator relationship matrix constructed based on the pedigree information, and $\mathbf{D}$ is a diagonal matrix containing “1s” for cows with phenotypes or ${\mathrm{n}}_{\mathrm{i}}$ for bulls with MACE DRP, where ${\mathrm{n}}_{\mathrm{i}}$ represents the MACE effective daughter contribution (EDC) [10]) for stature. ${\upsigma }_{\mathrm{a}}^{2}=5.50$ and ${\upsigma }_{\mathrm{e}}^{2}=4.63$ represent the additive polygenic and residual variance components, respectively. It should be noted that the variance components and the proportion of residual additive polygenic variance were not estimated, but were set to fixed values that corresponded to the parameters used in the Polish national genetic and genomic evaluation for stature.

### Convergence

The effects of this model were estimated using the MiXBLUP software [11] that implements the two-level PCG [5] method for solving the following system of equations:

2 $${{\mathbf{P}}^{-1}\mathbf{M}}^{-1}\mathbf{C}\mathbf{x}={{\mathbf{P}}^{-1}\mathbf{M}}^{-1}\mathbf{b},$$

where $\mathbf{C}$ is the coefficient matrix corresponding to the mixed model equations (MME) for solving Eq. (1), $\mathbf{x}$ is the vector of fixed and random effects given by ${\mathbf{x}}^{\mathrm{T}}=\left[{{\varvec{\upbeta}}}^{\mathrm{T}}{\mathbf{g}}^{\mathrm{T}}{\mathbf{u}}^{\mathrm{T}}\right]$, $\mathbf{b}$ is the right hand side (RHS) of the MME, and $\mathbf{M}$ and $\mathbf{P}$ are the first level and the second level preconditioning matrices, respectively.

In our study, the convergence rate of $\mathbf{x}$ was expressed by three criteria: CK, CM, and CD, which is the relative absolute difference over the whole $\mathbf{x}$. The CK criterion was proposed by [12] as: $\frac{1}{{\upmu }_{1}}\cdot \frac{\Vert {\mathbf{M}}^{-1}\left[\mathbf{b}-\mathbf{C}{\widehat{\mathbf{x}}}_{\mathbf{i}}\right]\Vert }{\Vert {\widehat{\mathbf{x}}}_{\mathbf{i}}\Vert }$, where ${\upmu }_{1}$ is the smallest active positive eigenvalue of the preconditioned coefficient matrix from Eq. (2) that influences the convergence and subscript i represents the round of iteration. The CM criterion was also proposed by [12] as: $\upkappa \left({\mathbf{M}}^{-1}\mathbf{C}\right)\cdot \frac{\Vert {\mathbf{M}}^{-1}\left[\mathbf{b}-\mathbf{C}{\widehat{\mathbf{x}}}_{\mathbf{i}}\right]\Vert }{\Vert {\mathbf{M}}^{-1}\mathbf{b}\Vert }$, where $\upkappa$(${\mathbf{M}}^{-1}\mathbf{C})$ is the effective spectral condition number of the ${\mathbf{M}}^{-1}\mathbf{C}$ matrix. The CD criterion is given by: $\frac{\Vert {\widehat{\mathbf{x}}}_{\mathbf{i}-1}-{\widehat{\mathbf{x}}}_{\mathbf{i}}\Vert }{\Vert {\widehat{\mathbf{x}}}_{\mathbf{i}}\Vert }$, where ${\widehat{\mathbf{x}}}_{\mathbf{i}}$ represents estimates from the ith iteration. In our study, $\mathrm{CD}\le 1\mathrm{e}-09$ was used as the stopping criterion, which indicates convergence of the equation system. The absolute difference is calculated as: $\left|{\widehat{\mathbf{x}}}_{\mathbf{i}}-{\widehat{\mathbf{x}}}_{\mathbf{F}}\right|$, where $\mathrm{F}$ corresponds to the estimates from the final iteration upon convergence. The absolute difference was calculated every 20th iteration, starting from the first iteration, separately for the following four groups of animals: (i) animals with genotypes and phenotypes $\left({\mathbf{G}}^{+}{\mathbf{P}}^{+}\right)$, including 59,242 animals, (ii) animals without genotypes and with phenotypes $\left({\mathbf{G}}^{-}{\mathbf{P}}^{+}\right)$, including 1,180,846 animals, (iii) animals with genotypes and without phenotypes $\left({\mathbf{G}}^{+}{\mathbf{P}}^{-}\right)$, including 75,718 animals, and (iv) animals without genotypes and phenotypes $\left({\mathbf{G}}^{-}{\mathbf{P}}^{-}\right)$, including 240,189 animals. Likewise, every 20th iteration, the absolute difference between estimated SNP effects was calculated.

The default stopping criterion ($\frac{\Vert {\widehat{\mathbf{x}}}_{\mathbf{i}-1}-{\widehat{\mathbf{x}}}_{\mathbf{i}}\Vert }{\Vert {\widehat{\mathbf{x}}}_{\mathbf{i}}\Vert }\le 1.0$E–09), implemented into the MiXBLUP software, was used for the termination of the optimisation process. The optimisation and the corresponding results presented below were run in parallel using 12 cores. In addition, to assess the potential numerical instability of PCG due to parallel computations, the FULL model was also evaluated in serial execution using a single core.

## Results

**Fig. 1:**
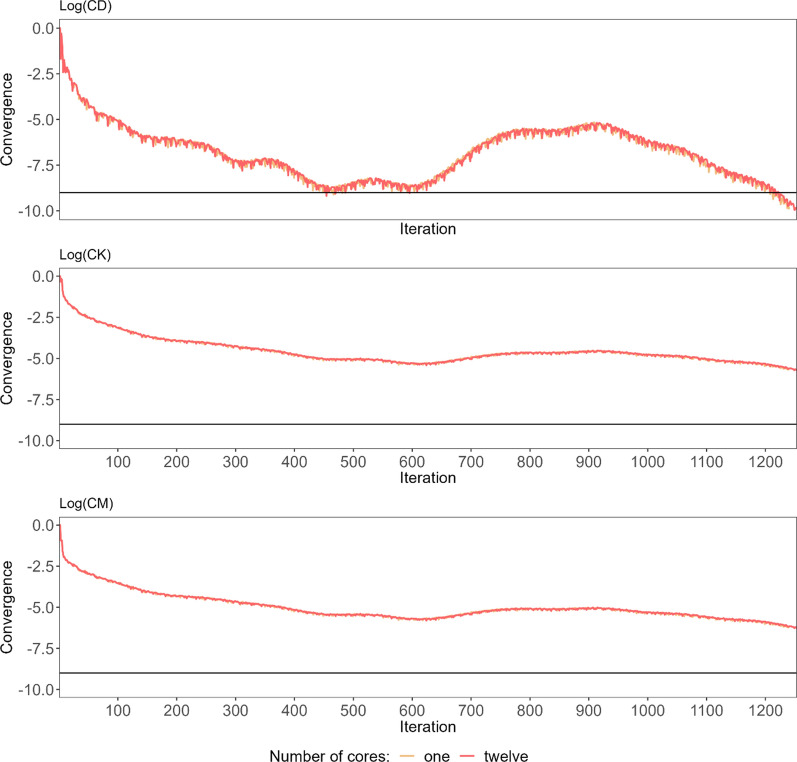
The convergence criteria (CK, CM, CD) of PCG along the optimisation process. The convergence criteria for one and 12 cores for the FULL dataset. The black line represents the stopping criterion defined by CD ≤ 1e−09

**Fig. 2:**
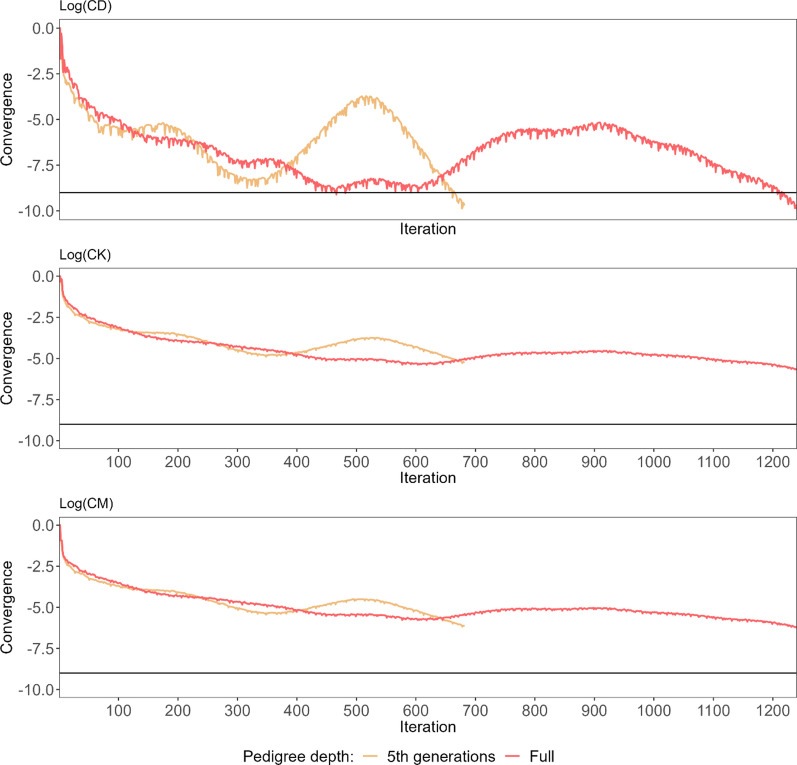
The convergence criteria (CK, CM, CD) of PCG along the optimisation process. The convergence criteria for the FULL and 5GEN datasets. The black line represents the stopping criterion defined by CD ≤ 1e−09

The convergence of the FULL dataset on 12 cores was achieved after 1240 iterations but when the model was run on a single core, it was reached 13 iterations later (Fig. 1). The 5GEN dataset with 682 iterations converged twice as fast (Fig. 2). Both convergence measures were very similar and demonstrated a nonlinear, even nonmonotonical, improvement with an increase in CK and CM before reaching the final convergence. Pearson correlations between estimated breeding values (EBV) predicted based on those two datasets were very close to 1 regardless of the sex and animal group considered. As expected, the lowest correlations of 0.946 (bulls) and 0.980 (cows) were estimated for the least informative $\left({\mathbf{G}}^{-}{\mathbf{P}}^{-}\right)$ group (Table 2).

**Table 2:** Correlations between EBV predicted based on the full pedigree and on the pedigree truncated after the 5th generation for different groups of animals

Group of animals	Number of animals	Correlation	
		Males	Females
All	1,555,995	0.997	0.991
Phenotype and genotype	59,242	0.999	0.999
Only phenotype	1,180,846	0.999	0.999
Only genotype	75,718	0.999	0.999
Without genotype and phenotype	240,189	0.946	0.980

**Fig. 3:**
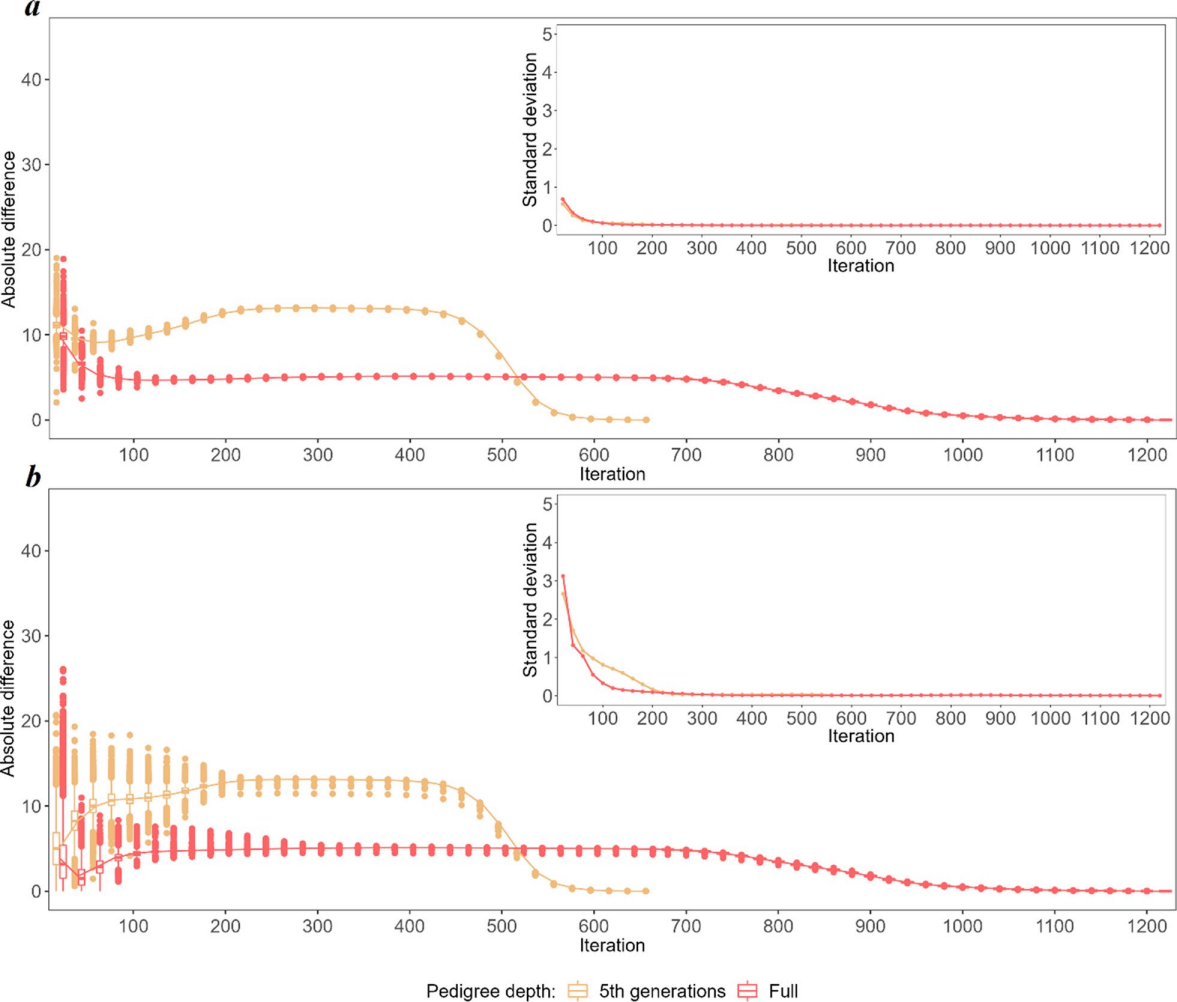
The average absolute difference in estimated breeding values (EBV) between a given round of iteration and the final solution (main graph) and their standard deviations (inside graph) during the optimisation process for animals with phenotypes and genotypes $\left({\mathrm{G}}^{+}{\mathrm{P}}^{+}\right)$. **a** Shows the convergence criteria for the FULL and 5GEN datasets. **b** Shows the convergence criteria for one and 12 cores for the FULL dataset

To explore the non-linearity of the convergence criteria in detail, the predicted breeding values (BV) and SNP effects obtained in each of the considered iterations were compared with their final values (Figs. 3 and 8). The three most striking features of the iterative process, regardless of the category considered, were (i) the convergence was reached twice as fast for the 5GEN than for the FULL dataset, (ii) as a result, the patterns of convergence expressed by the variability of some predicted values and by their differences to the final solution are much more pronounced in the 5GEN than in the FULL dataset, (iii) however, during iterations, before reaching convergence, the FULL dataset always resulted in estimates being considerably more similar to their final solutions than the 5GEN set, which may be due to differences in the preconditioning matrices $\mathbf{M}$ between both datasets. Regardless of the magnitude of the initial difference between EBV estimates in some iterations and the final estimate recorded for different groups, the convergence pattern is the same. After the initial phase showing a large variability in individual estimates, i.e. ~ the first 200 iterations, which sometimes even resulted in a relatively small averaged difference, the stable phase expressed by small differences in the accuracy of estimates across iterations was reached, followed by a rapid (5GEN) or monotonously decreasing (FULL) difference until the final estimate.

**Fig. 4:**
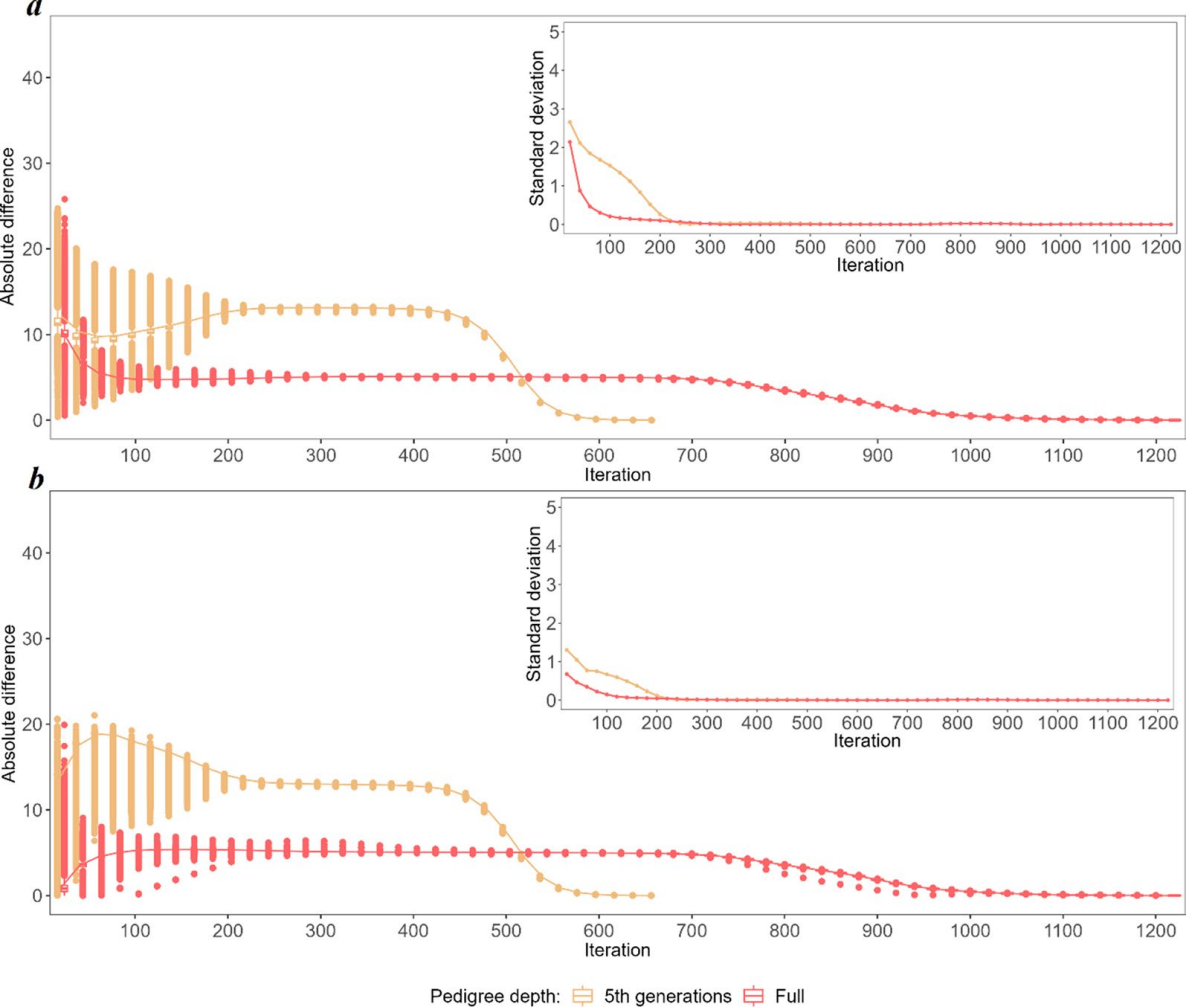
The average absolute difference in estimated breeding values (EBV) between a given round of iteration and the final solution (main graph) and their standard deviations (inside graph) during the optimisation process for ungenotyped animals with phenotypes $\left({\mathrm{G}}^{-}{\mathrm{P}}^{+}\right)$. **a** Represents bulls and **b** Represents cows

**Fig. 5:**
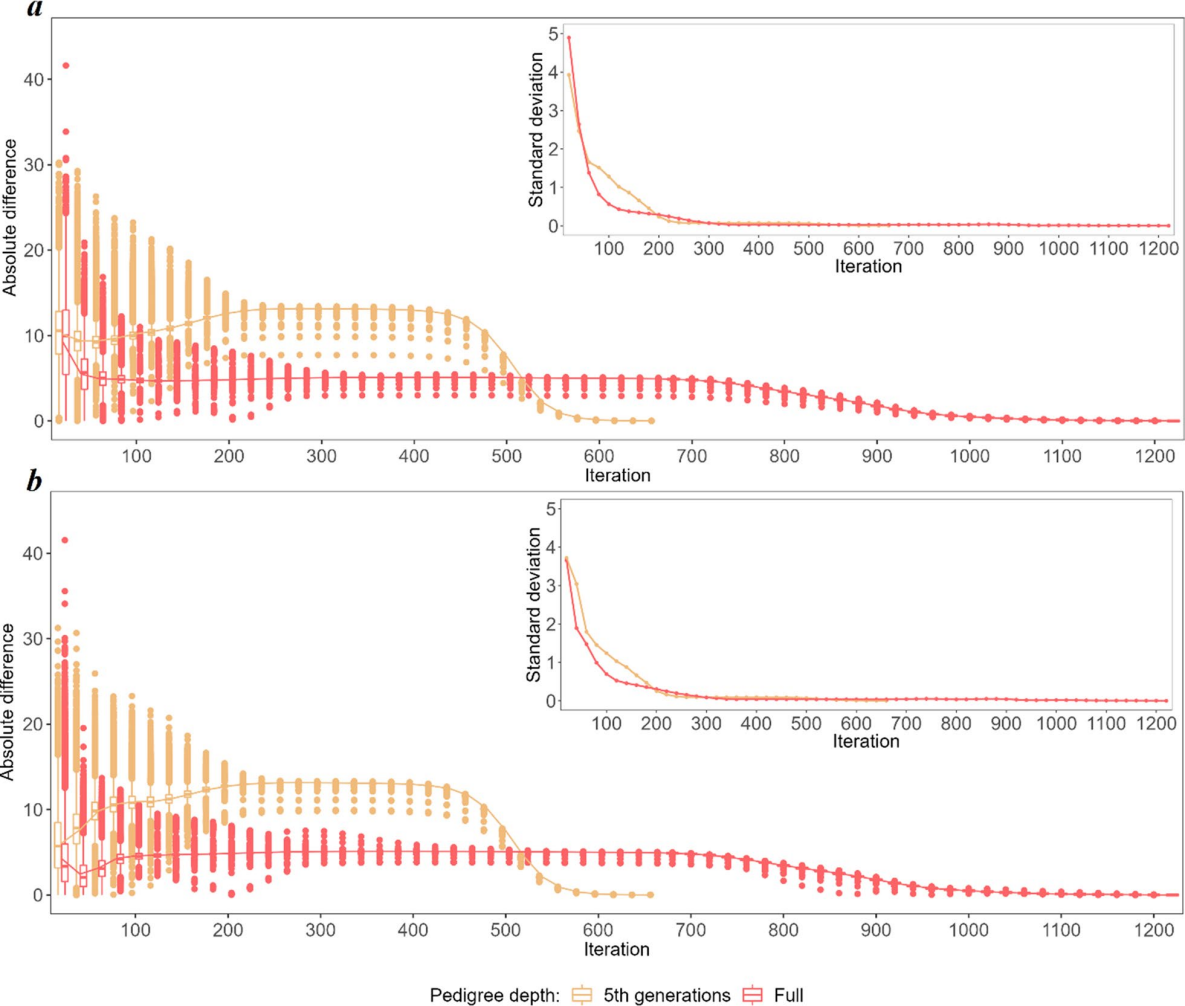
The average absolute difference in estimated breeding values (EBV) boxplots between a given round of iteration and the final solution (main graph) and their standard deviations (inside graph) during the optimisation process for genotyped animals without phenotypes $\left({\mathrm{G}}^{+}{\mathrm{P}}^{-}\right)$. **a** Represents bulls, and **b** Represents cows

**Fig. 6:**
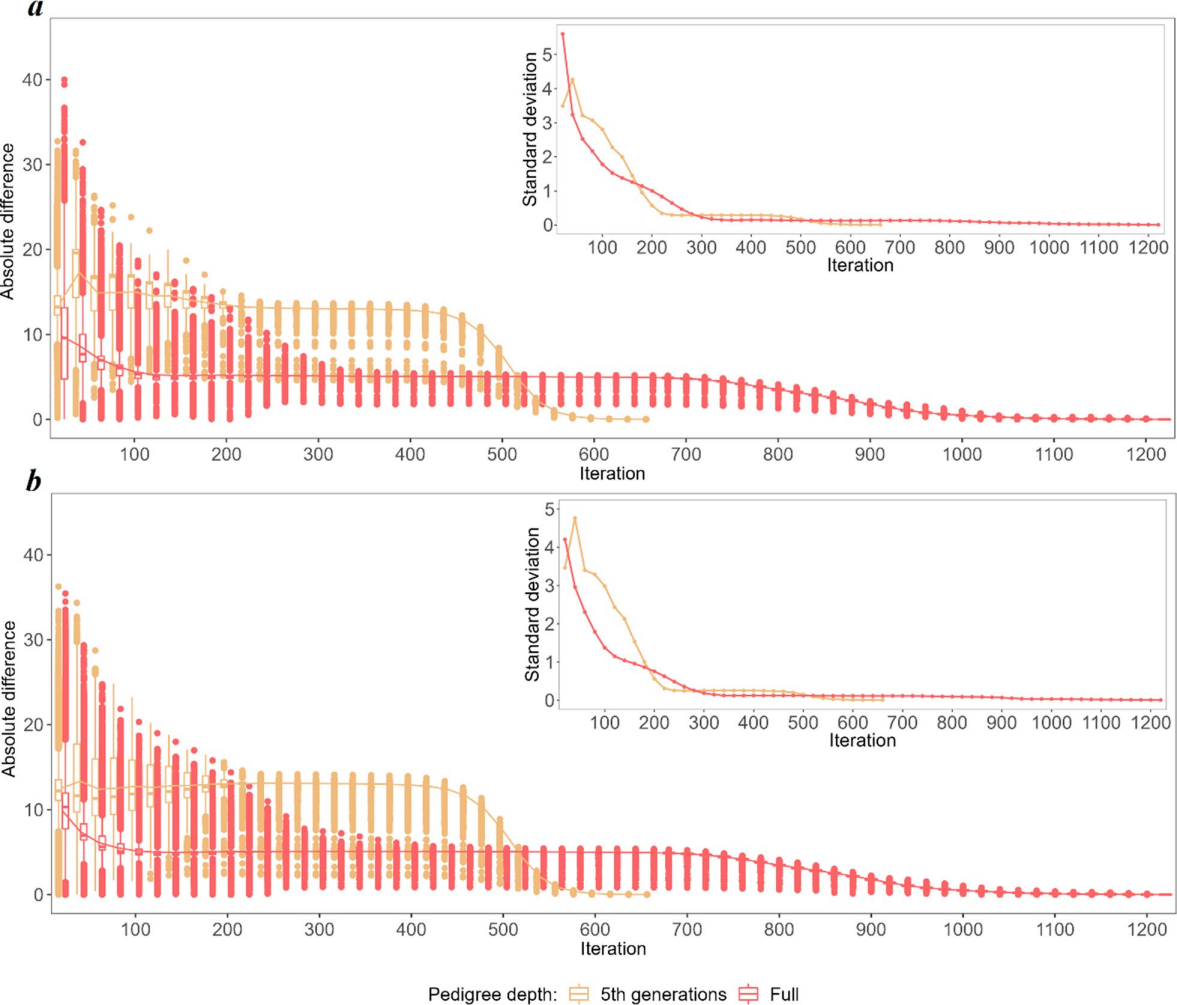
The average absolute difference in estimated breeding values (EBV) between a given round of iteration and the final solution (main graph) and their standard deviations (inside graph) during the optimisation process for ungenotyped animals without phenotypes $\left({\mathrm{G}}^{-}{\mathrm{P}}^{-}\right)$. **a** Represents bulls, and **b** Represents cows

**Fig. 7:**
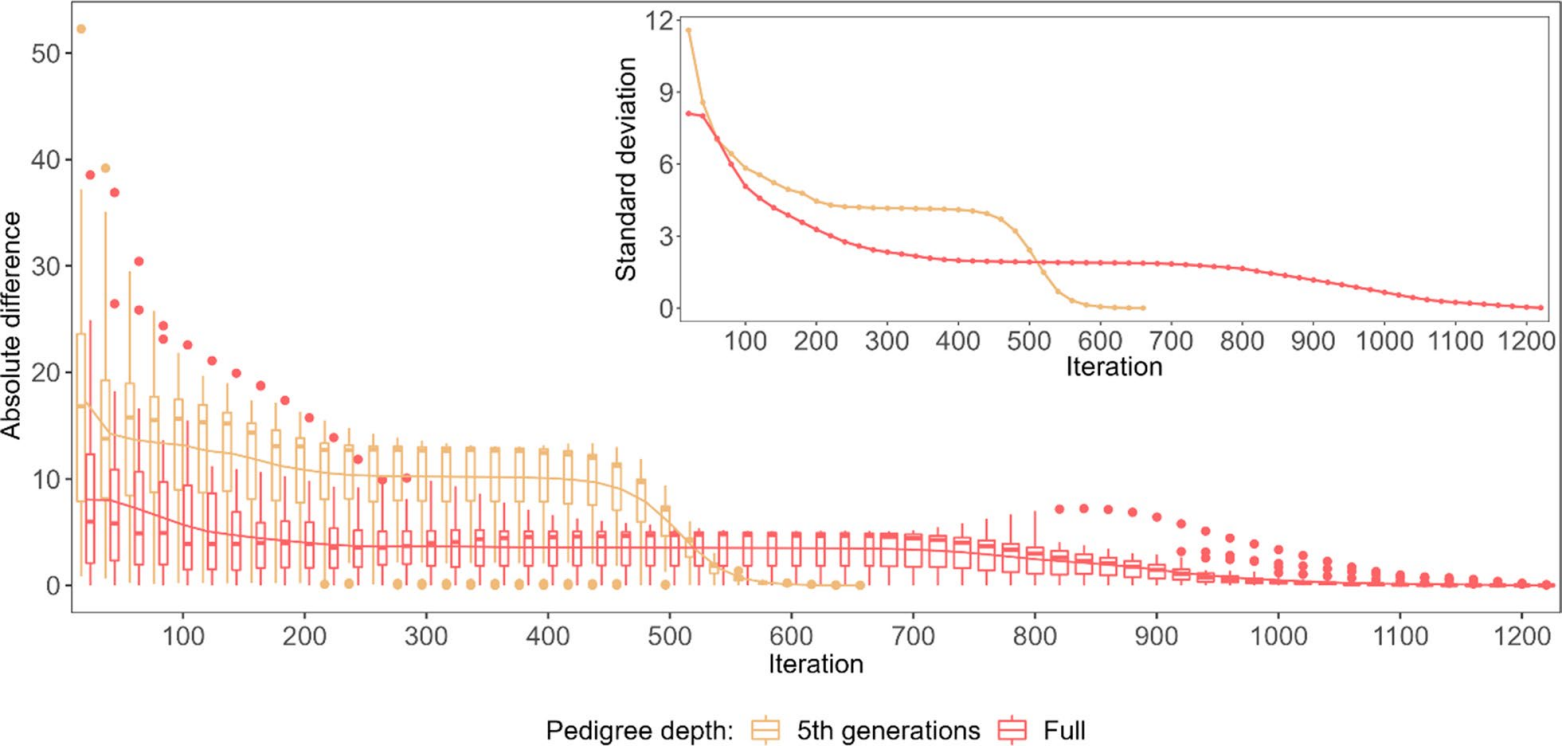
The average absolute difference in the estimates of genetic groups effects between a given round of iteration and the final solution (main graph) and their standard deviations (inside graph) during the optimisation process

**Fig. 8:**
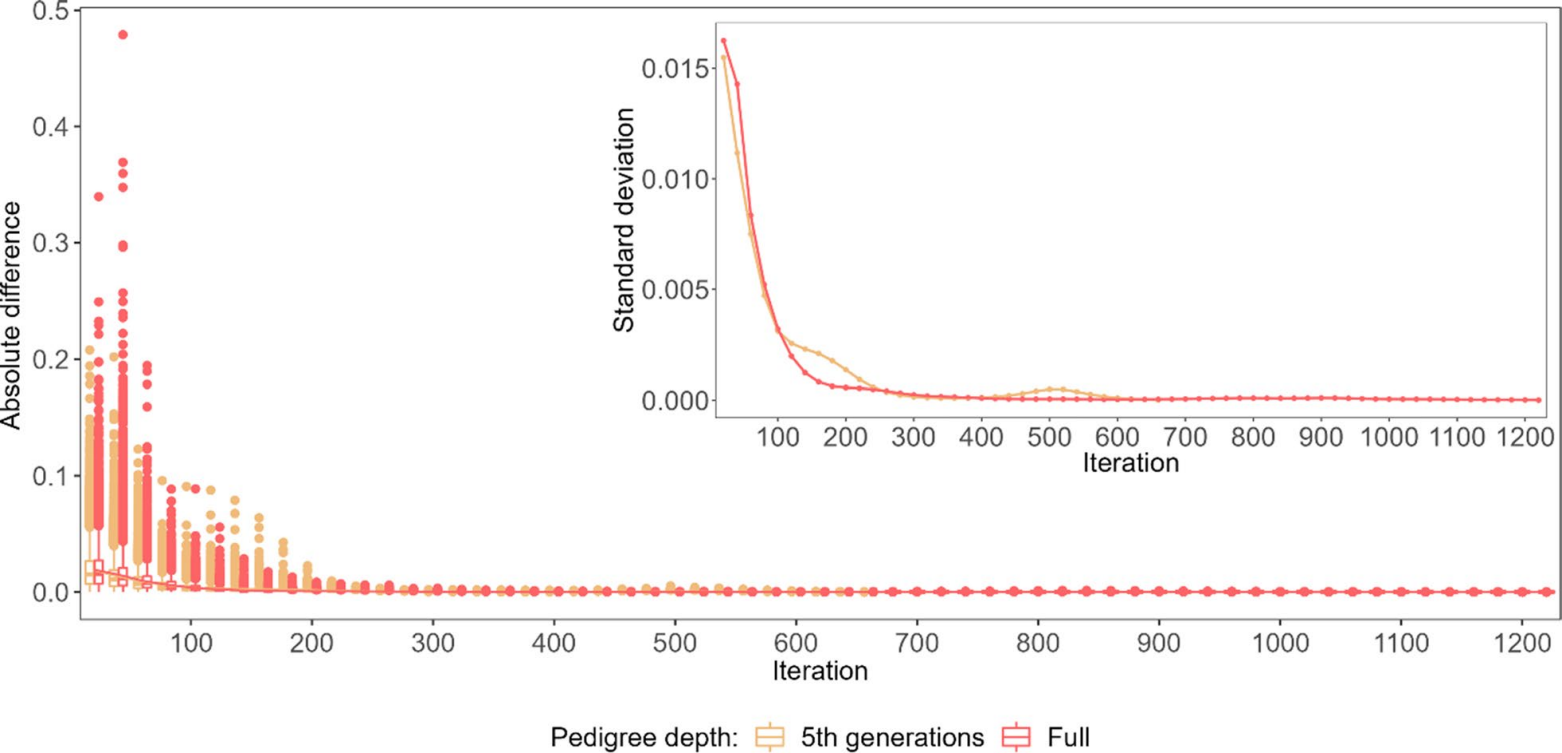
The average absolute difference in the estimates of SNP effects between the particular iteration and the final solution (main graph) and their standard deviations (inside graph) during the optimisation process

**Fig. 9:**
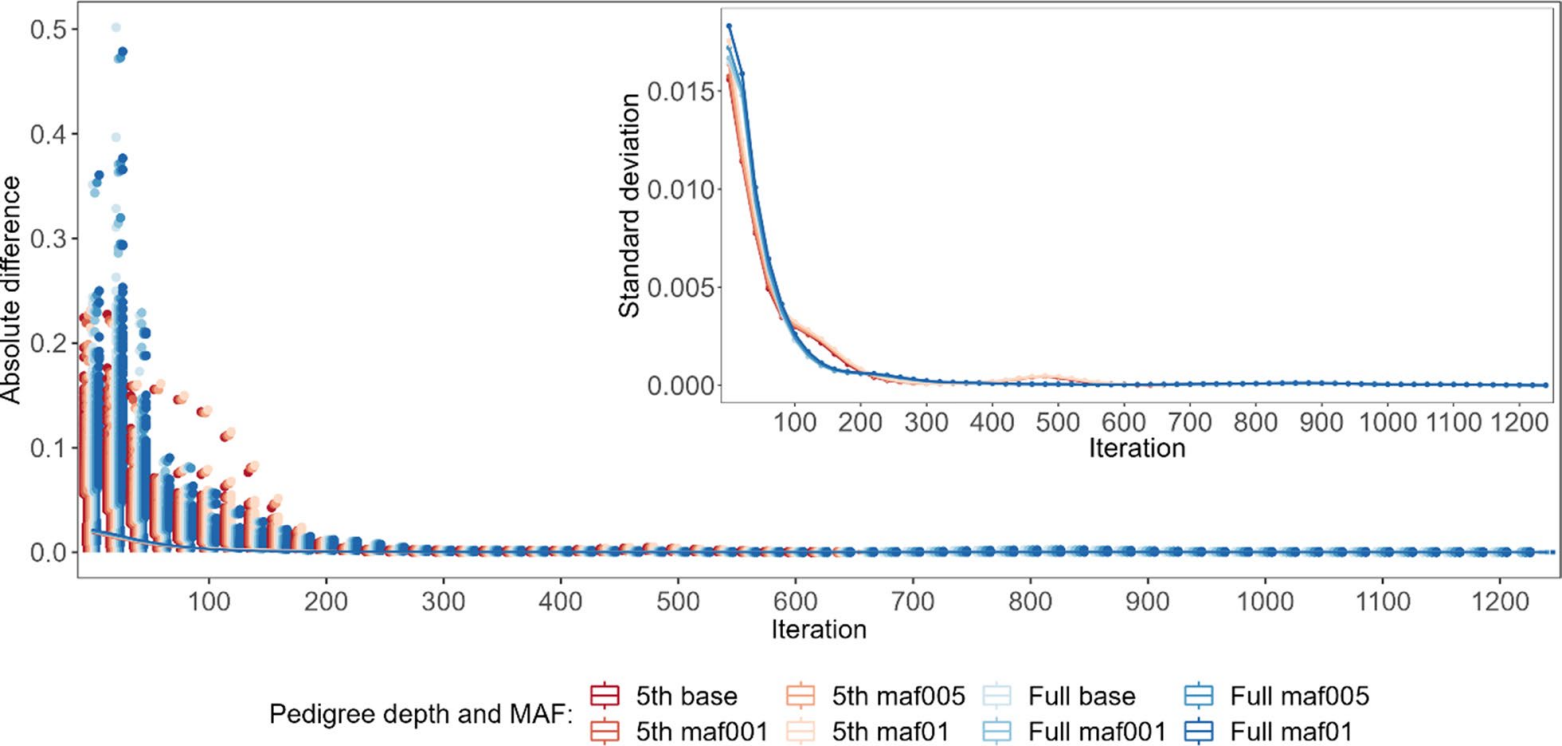
The average absolute difference in the estimates of SNP effects between the particular iteration and the final solution (main graph) and their standard deviations (inside graph) during the optimisation process for different SNP preselection criteria based on the MAF threshold

Considering the specific components of the random effects’ solution vector, the EBV of animals with both sources of information available $\left({\mathbf{G}}^{+}{\mathbf{P}}^{+}\right)$ demonstrated the smallest absolute differences between the final EBV and the EBV estimated during the optimisation iterations; in addition, their standard deviations (especially for the bulls) were the smallest of all considered groups of animals, which may indicate good quality starting values for the iteration process (Fig. 3) and higher reliabilities of the EBV of the animals as compared to the other groups. For the $\left({\mathbf{G}}^{-}{\mathbf{P}}^{+}\right)$ group, the pattern and average absolute difference in EBV convergence were very similar to those of the $\left({\mathbf{G}}^{+}{\mathbf{P}}^{+}\right)$ group, but a much larger variability of some predicted EBV was observed for bulls (Fig. 4). The same results were found for the $\left({\mathbf{G}}^{+}{\mathbf{P}}^{-}\right)$ group (Fig. 5). In spite of the similar convergence pattern as expressed by the average absolute difference, during almost the full process of iterations a considerably larger variability in the predicted EBV was observed for some individuals from the least informative $\left({\mathbf{G}}^{-}{\mathbf{P}}^{-}\right)$ group. For this group, the EBV reliabilities were the lowest among the four groups, which indicates that certain members of this group are the limiting factor that affects convergence (Fig. 6). Unlike the EBV, the estimation of the effects of genetic groups revealed a monotonically improving pattern of convergence. However, the most striking feature was the heterogeneity in convergence behaviour between the two datasets and some groups. Although the estimates of some genetic groups of the 5GEN dataset already reached convergence after the 200th iteration, for the FULL dataset the convergence of the estimates was poor (Fig. 7). The opposite convergence behaviour was attributed to SNP effects that, regardless of the dataset, converged very fast, so that final estimates were already available at the 300th iteration, i.e. in the middle (5GEN) or even at one-third (FULL) of the whole optimisation process (Fig. 8). The correlation between the final estimates upon convergence of the FULL dataset resulting from parallel and linear computations was 0.99, with some differences occurring from the third decimal place. Conversely, the four SNP subsets demonstrated very similar convergence rates and patterns (Fig. 9).

Finally, we compared the 50 top EBV ranking bulls between the 5GEN and FULL datasets and found 49 overlapping bulls. The single bull from the 5GEN data that was not in the top 50 bulls of the FULL dataset was still classified at the relatively high 52nd rank. In contrast, one bull that ranked high in the FULL dataset was missing in the top 50 bulls in 5GEN dataset, this bull was not evaluated in Poland, and its high predicted EBV in the FULL dataset was due to several highly ranked relatives that were present in the full pedigree records. The overall rank correlation for the remaining 49 individuals was 0.99.

## Discussion

The most striking result of our study was the much faster convergence of the reduced (5GEN) dataset than that of the full dataset, although solutions from the initial rounds of iterations were much better (i.e. closer to the final solutions) for the FULL dataset. Although in our study, this result was obtained by solving a single-step SNP-BLUP model, Pocrnic et al. [13] reported the same result with the single-step genomic BLUP (GBLUP) model. In addition, Legarra et al. [1] observed potential convergence problems for data structures composed of a deep pedigree with many generations of ungenotyped individuals. Another common feature of the solving process was a non-linear and even non-monotonic convergence pattern. Although Vandenplas et al. [12] indicated an approximately linear convergence expressed by the CK, CM, and CD criteria, our study as well as other applications of PCG to the solving of mixed linear models in the context of SNP-BLUP and GBLUP [4, 13–16] reported a pattern that approximates a typical nonlinear behaviour that involves an initial phase of fast convergence rate, a second phase characterised by an almost linear convergence rate, and a third fast converging phase, although with a non-monotonic decrease of the convergence measures. However, for most of the considered scenarios, the initial phase did not always show a rapid linear convergence of estimates, as expressed by their absolute difference from the final solution, and involved a small number of iterations. Furthermore, we observed differences in the initial convergence rate that varied not only between the two datasets (FULL vs. 5GEN) but also between the groups of effects considered (EBV for ${\mathbf{G}}^{+}{\mathbf{P}}^{+}$, ${\mathbf{G}}^{+}{\mathbf{P}}^{-}$, ${\mathbf{G}}^{-}{\mathbf{P}}^{+}$, ${\mathbf{G}}^{-}{\mathbf{P}}^{-}$; genetic groups, and SNPs). We hypothesise that the differences between datasets are due to the pedigree structure, which results in a smaller number of individuals without genotype and phenotype information. Furthermore, the differences between animal groups are due to different information contents that are implemented into the preconditioner matrix $\mathbf{M}$ and that impact the quality of preconditioning, as defined in [5], or to the effects of genetic groups and breeding values of the ${\mathbf{G}}^{-}{\mathbf{P}}^{-}$ individuals, which are predicted only indirectly based on relatives with genotypic and/or phenotypic data. An interesting convergence pattern was observed when comparing the FULL and the 5GEN datasets, i.e. the second (linear) convergence phase was always much longer with the FULL dataset, which may result from the fact that equation systems of large dimensions are typically not as well conditioned as smaller equation systems, which impacts the efficiency of iterative solvers [17]. Indeed, in the case of our data, the condition number, computed as the ratio of the approximated extremal eigenvalues, was twice as high for the FULL dataset (4,258 285) than for the 5GENE dataset (2,171,642), resulting in a smaller effective spectral condition number that can be related with a faster convergence. In addition, numerical instability, which is due to rounding errors since the evaluation of a complete pedigree involves many more arithmetic operations, may further hamper the numerical performance [4, 6].

In the original application of the PCG algorithm for the optimisation of the single-step BLUP evaluation, it was observed that SNP effects posed a problem for the efficient convergence of the solver [4]. However, in the current evaluation, among all the effects, SNP estimates demonstrated the fastest convergence from the beginning of the iteration and then, by far, the fastest and nearly linear convergence towards the final solutions. On the one hand, this is due to the implementation of an additional pre-conditioning matrix (the second-level preconditioner $\mathbf{P}$) into the current version of the MiXBLUP software. On the other hand, a smaller number of genotyped individuals (compared to the number of phenotyped individuals) available for the estimation of SNP effects corresponds to a smaller number of arithmetic operations involved, which implies a smaller number of rounding errors by storing and processing real type variables involved in the computations [4]. The third observation was that the faster convergence of SNP effects may also be due to more information content being available for these in the dataset, since the EBV of all genotyped animals make contributions to the estimation of SNP effects.

Finally, we analysed potential convergence problems that can originate from the additional numerical complexity imposed due to the implementation of parallel computations, as indicated by [6]. However, this phenomenon was not observed in the single-step SNP-BLUP implementation.

## Conclusions

Our findings demonstrate that not all effects estimated in the single-step SNP-BLUP model have the same convergence rate. The effects of SNPs converged the fastest, whereas those of the genetic groups had the lowest rate of convergence. Among the four groups of animals, the EBV of the genotyped animals with phenotype data reached the final solutions with the smallest number of iterations, whereas the nongenotyped animals without own phenotype records required the largest number of rounds of iterations to reach their final solutions. The depth of pedigree markedly influences the rate of convergence, with fewer generations in the pedigree leading to faster convergence. We believe, that the observed convergence patterns are not specific to the dataset analyzed here, which reflects a structure of a standard national dairy population, but that they also apply to other national populations.

## Data Availability

The data set corresponding to the genetic evaluation from December 2021 can be obtained from the Institute of Animal Breeding on request to ocena.bydlo@iz.edu.pl.
